# Protective Role of Short-Chain Fatty Acids against Ang- II-Induced Mitochondrial Dysfunction in Brain Endothelial Cells: A Potential Role of Heme Oxygenase 2

**DOI:** 10.3390/antiox12010160

**Published:** 2023-01-10

**Authors:** Modar Kassan, Youngin Kwon, Undral Munkhsaikhan, Amal M. Sahyoun, Tauheed Ishrat, María Galán, Alexis A. Gonzalez, Ammaar H. Abidi, Adam Kassan, Karima Ait-Aissa

**Affiliations:** 1 College of Dental Medicine, Lincoln Memorial University, Knoxville, TN 37917, USA; 2 Department of Physiology, University of Tennessee Health Science Center, Memphis, TN 38163, USA; 3 Department of Bioscience Research and General Dentistry, College of Dentistry, The University of Tennessee Health Science Center, Memphis, TN 38163, USA; 4 Department of Food Science and Agriculture Chemistry, McGill University, Montreal, QC H9X 3V9, Canada; 5 Neuroscience Institute, University of Tennessee Health Science Center, Memphis, TN 38163, USA; 6 Department of Basic Sciences of Health, Area of Biochemistry and Molecular Biology, University Rey Juan Carlos, Centro de Investigación Biomédica en Red de Enfermedades Cardiovasculares (CIBERCV), ISCIII, 28922 Madrid, Spain; 7 Instituto de Química, Pontificia Universidad Católica de Valparaíso, Valparaíso 2340000, Chile; 8 Department of Pharmaceutical Sciences, School of Pharmacy, West Coast University, Los Angeles, CA 90004, USA; 9 Cardiovascular Division, Department of Medicine, Abboud Cardiovascular Research Center, Carver College of Medicine, University of Iowa, Iowa City, IA 52242, USA

**Keywords:** mitochondrial dysfunction, heme oxygenase 2, SCFAs, brain endothelial cells

## Abstract

Objectives: Short-chain fatty acids (SCFAs), the main metabolites released from the gut microbiota, are altered during hypertension and obesity. SCFAs play a beneficial role in the cardiovascular system. However, the effect of SCFAs on cerebrovascular endothelial cells is yet to be uncovered. In this study, we use brain endothelial cells to investigate the in vitro effect of SCFAs on heme oxygenase 2 (HO-2) and mitochondrial function after angiotensin II (Ang-II) treatment. Methods: Brain human microvascular endothelial cells were treated with Ang-II (500 nM for 24 h) in the presence and absence of an SCFAs cocktail (1 μM; acetate, propionate, and butyrate) and/or HO-2 inhibitor (SnPP 5 μM). At the end of the treatment, HO-2, endothelial markers (p-eNOS and NO production), inflammatory markers (TNFα, NFκB-p50, and -p65), calcium homeostasis, mitochondrial membrane potential, mitochondrial ROS and H_2_O_2_, and mitochondrial respiration were determined in all groups of treated cells. Key Results: Our data showed that SCFAs rescued HO-2 after Ang-II treatment. Additionally, SCFAs rescued Ang-II-induced eNOS reduction and mitochondrial membrane potential impairment and mitochondrial respiration damage. On the other hand, SCFAs reduced Ang-II-induced inflammation, calcium dysregulation, mitochondrial ROS, and H_2_O_2_. All of the beneficial effects of SCFAs on endothelial cells and mitochondrial function occurred through HO-2. Conclusions: SCFAs treatment restored endothelial cells and mitochondrial function following Ang-II-induced oxidative stress. SCFAs exert these beneficial effects by acting on HO-2. Our results are opening the door for more studies to investigate the effect the of SCFAs/HO-2 axis on hypertension and obesity-induced cerebrovascular diseases.

## 1. Introduction

Mitochondria play an important role in cellular respiration, cell death, the regulation of innate immunity, and calcium homeostasis, and are crucial in regulating brain microvascular function and cerebral blood flow [1,2]. Interestingly, the mitochondria-mediated vascular tone in cerebral arteries is disrupted during hypertension [3] and obesity [4]. Thus, the direct or indirect regulation of mitochondria function during hypertension might prove to be an effective treatment in the prevention of cerebrovascular diseases.

Short-chain fatty acids (SCFAs) are the main metabolites produced by the fermentation of dietary fibers by the colonic microbiota. SCFAs play a key role in regulating mitochondrial function and levels are known to be reduced during hypertension [5]. They also play an important role in regulating obesity [6]. The effects of SCFAs are not restricted to the intestine, as they are small molecules that can diffuse through gut enterocytes, enter blood circulation, and participate in peripheral tissue metabolism [7]. In the systemic circulation, SCFAs affect energy homeostasis and metabolism by regulating mitochondrial functions and dynamics in brown adipocytes, liver, and skeletal muscle via G protein-coupled receptor (GPR) 41, GPR 43, and the free fatty acid receptor (FFAR) [8]. While SCFAs are known to cross the blood–brain barrier and affect the central nervous system [9], little is known about their effect on cerebral blood flow (CBF) and brain microvascular function. Brain microvascular endothelial cells (BMEC) are known to express the SCFAs receptors GPR 41, GPR 43, and FFAR, indicating that SCFAs might be involved in BMEC activity and mitochondrial function [10]. 

Heme oxygenase (HO) enzymes, the rate-limiting enzymes in the breakdown of heme, are critical for maintaining cellular homeostasis [11]. Two isoforms have been reported to date: HO-1 and HO-2. The HO-1 isoform has been extensively studied in endothelial cells and other tissues mainly because of its ability to respond to cellular stresses such as nitric oxide donors, oxidative damage, hypoxia, and others [12]. Numerous studies have reported that HO-1 translocates to the mitochondria and regulates mitochondrial function. Indeed, HO-1 acts through calcium channels (such as the Mitochondrial Calcium Uniporter channel) and peroxisome proliferator-activated receptor-gamma coactivator (PGC)-1alpha to regulate mitochondrial biogenesis, oxygen consumption, adenosine triphosphate (ATP) production, and electron transport chain activity to produce cytoprotective effects [13,14]. By contrast, due to its constitutive nature, the focus on HO-2 has been limited. Nevertheless, its abundance in endothelial cells, particularly in the brain, has pointed toward the potential relevance of HO-2 involvement in brain vascular function [15]. Thus, the relationship between HO-1 and mitochondrial function is well-documented; however, less is known about the involvement of HO-2 in mitochondrial function, especially in brain endothelial cells. Recently, a study showed that similar to HO-1, HO-2 can translocate to the mitochondria [16]. However, the study did not offer a molecular mechanism for this translocation.

Our preliminary data showed that during oxidative stress conditions, SCFAs are not only able to rescue HO-2 activity but also restore mitochondrial function through HO-2 signaling pathways. The outcome of this study will open the door for the identification of novel targets for the prevention and/or treatment of cerebrovascular events during hypertension and obesity.

## 2. Materials and Methods

### 2.1. Reagents 

The SCFAs cocktail components of sodium acetate (#S2889), sodium butyrate (#303410), and sodium propionate (#P5436) were obtained from Sigma-Aldrich. HO-2 inhibitor, tin protoporphyrin IX (chloride), and (#16375) were purchased from Cayman Chemical. Angiotensin II (Ang-II, #A2900) was obtained from Sigma. Tetramethylrhodamine methyl ester (TMRM, #T668) was purchased from Molecular Probes. MitoSOX Red (#D1168), MitoTracker Green FM (#M7514), and 4,5-Diaminofluorescein diacetate (DAF2-DA; #D23844) were obtained from ThermoFisher, Waltham, MA, USA; Nomega-Nitro-L-arginine (L-NNA, #ab141312) and sodium nitroprusside (SNP, #S-0501) were purchased from Abcam and Sigma, respectively. MitoTEMPO (#SML0737) and Rotenone (#R8875) were purchased from Sigma-Aldrich. Human brain microvascular endothelial cells (HBMECs, #1000) and Endothelial Cell Medium (ECM) supplemented with growth factors (#1001) were obtained from ScienCell. The following antibodies were obtained from Cell Signaling Technology and used for immunoblotting: anti-phosphorylated (p-) endothelial nitric oxide synthase (eNOS) (#9571), anti-total (t-) eNOS (#9572), and anti-GAPDH (#2178). The anti-HO-2 antibody (#ADI-OSA-200-D) was purchased from Enzo. 

### 2.2. Endothelial Cell Culture 

Primary HBMECs were grown in endothelial cell medium (ECM) at 37 °C and 5% CO_2_ and used at passages 3–5. At confluency, cells were treated with angiotensin (Ang) -II (500 nM for 24 h) in the presence or absence of a cocktail of SCFAs (sodium acetate, sodium butyrate, and sodium propionate) at a dose of 1 μM for 24 h. For HO-2 inhibition, HBMECs were treated with 5 μM tin protoporphyrin IX in dimethylformamide (DMF) for 24 h. Control cells were treated with DMF.

### 2.3. Heme Oxygenase Activity Assay

Heme oxygenase activity was analyzed using a commercially available bilirubin assay according to the manufacturer’s instructions (MyBioSource). Briefly, following treatments, HBMECs were harvested, homogenized in cold PBS, and centrifuged at 10,000× *g* for 10 min at 4 ºC. After centrifugation, the supernatant containing the whole-cell proteins was quantified using the BCA assay (ThermoFisher) and processed for the bilirubin assay kit. The relative activity was quantified using a generated standard curve.

### 2.4. Protein Expression

Western blot analysis for HO-2, t- and p-eNOS was performed in cell lysates as previously described [17,18]. Briefly, the cells were harvested, lysed in RIPA buffer supplemented with proteinase and phosphatase inhibitors, and then sonicated using a sonicator. After centrifugation for 10 min at 10,000 rpm, the total protein was quantified using the BCA assay and 20 ug of protein per sample were loaded into SDS-PAGE gels. The proteins were then transferred to PVDF membranes, incubated in 5% milk, and incubated with primary antibodies for HO-2 (1:1000 dilution), total- and phosphorylated eNOS (1:1000 dilution). GAPDH (1:5000) was used as a loading control. 

### 2.5. Measurement of Ca^2+^ Uptake by Mitochondria 

Ratiometric measurements of [Ca^2+^] in mitochondria were performed in HBMECs using mitochondrial Ca^2+^ adenovirus (_mt_)Pericam (Ad-_mt_Pericam), as previously described [19]. Pericam fluorescence was detected using a customized Nikon Eclipse Ti2 inverted light microscope. Pericam was excited at 405 nm and 480 nm, and its emission was recorded at 535 nm. Real-time Pericam fluorescence ratios were recorded before and after platelet-derived growth factor (PDGF) was added (20 ng/mL) and were quantified using ImageJ. The summary data represent the average difference in basal mitochondrial [Ca^2+^].

### 2.6. Measurement of Cytosolic Ca^2+^

Calcium release into the cytosol was measured, as previously described [20]. Briefly, pretreated primary HBMECs were loaded with 20 μM Fluo-4 for 15 min. Cells were then washed with isotonic buffer and the assay was performed in a buffer solution. Fluo-4 fluorescence was determined using a fluorescence microscope.

### 2.7. Measurement of Mitochondrial ROS Production

Mitochondrial ROS production was measured in live cultured HBMECs using the dihydroethidium derivative MitoSOX Red. Following treatments, cultured cells were rinsed in warm HBSS buffer and then loaded with MitoSOX Red (5 μM) and MitoTracker Green FM (1 μM) [21] diluted in HBSS buffer for 20 min at 37 °C. The cells were then rinsed in warm HBSS buffer, imaged using a fluorescence microscope, and analyzed using NIH ImageJ. The data are presented as the ratio of integrated density MitoSOX Red signal to MitoTracker Green FM signal. Cells were treated with Rotenone (1 μM) for 1 h and were used as a positive control. Other cultured HBMECs were pretreated with a mitochondrial ROS scavenger (mitoTEMPO, 10 μM) in the absence or presence of Ang-II. The cells were then washed and loaded with MitoTracker and MitoSOX following the same protocol as above. 

### 2.8. Measurement of Cellular Hydrogen Peroxide

To assess the H_2_O_2_ release in response to Ang-II in the presence or absence of SCFAs, extracellular H_2_O_2_ levels were measured using the fluorescent probe Amplex Red (Molecular Probes, ThermoFisher) [22] following the manufacturer’s instructions. Briefly, following treatment, cultured HBMECs were exposed to Amplex Red (2 mmol/L) diluted in the appropriate buffer. The supernatant was then collected and a volume of 100 µl was loaded into a 96-well plate and fluorescence was measured relative to standard controls generated by serial dilutions of H_2_O_2_ on a spectrophotometer using excitation and emission levels of 490 nm and 585 nm, respectively. To correct for background fluorescence, measurements were compared to a no-H_2_O_2_ control. All fluorescence values were normalized to the total protein from each dish using a BCA protein assay.

### 2.9. Quantification of Nitric Oxide

Nitric Oxide (NO) levels were measured using the fluorescent probe DAF2-DA (Sigma) [23]. Following treatments, cultured HBMECs were washed with warm DPBS and then incubated with a fluorescent nitric oxide probe and DAF2-DA (5 µM in ECM medium) for 60 min. The HBMECs were then rinsed and imaged under a fluorescence microscope. To induce NO release, HBMECs were exposed to PDGF (20 ng/mL) + glutamine (1 µM) [24]. Continuous imaging was performed for 10 min following stimulation. For the negative control measurements, the cells were subjected to LNNA for 30 min, then washed, and then assayed for NO production following the same protocol as above. For the positive control measurements, the DAF2-DA-loaded cells were stimulated with sodium nitroprusside (SNP). The amount of NO produced is expressed as fluorescence intensity normalized to that at baseline. 

### 2.10. Quantitative Real Time PCR

Total RNA from cultured cells was extracted using the RNeasy kit (Qiagen) following the manufacturer’s instructions. Quantitative real-time PCR was performed using a ViiA 7 Real-Time PCR System (Applied Biosystems, Foster City, CA, USA) with the SuperScript III Platinum SYBR Green One-Step qRT-PCR Kit (Invitrogen). The following primers purchased from Integrated DNA Technologies were used: vascular cell adhesion molecule 1 (VCAM1) (NM_001078.4) forward 5′- TGA CGA TGA CGT GTG CCA GT-3′, reverse 5′- GCT GTC GGT TCC CAT TGT CT-3′; nuclear factor-κB (NFκB) subunit p50 (NM_001382626.1) forward 5′- TGGACAGCAAATCCGCCCTG-3′, reverse 5′-TGTTGTAATGAGTCGTCATCCT-3′; NFκB subunit p65 (NM_001382626.1) forward 5′-AGGCAAGGAATAATGCTGTCCTG -3′, reverse 5′- ATCATTCTCTAGTGTCTGGTTGG -3′; ribosomal RNA 18S (NR_003278.3) forward 5′- CCCTATCAACTTTCGATGGTAGTCG -3′, reverse 5′-CCAATGGATCCTCGTTAAAGGATTT -3′, tumor necrosis factor-alpha (TNFα) (NM_000594.4) forward 5′-CACTAAGAATTCAAACTGGGGC-3′, TNFα reverse 5′- GAGGAAGGCCTAAGGTCCAC -3′. Ribosomal RNA 18S was used as an internal gene control.

### 2.11. Bioenergetics by Seahorse

For experiments in the Seahorse XF analyzer (Seahorse Bioscience), HBMECs were plated into 96-well Seahorse V3 PET plates at a density of 50,000 per well 24 h before the treatment. HBMECs were then washed and equilibrated in Seahorse assay medium containing 25 mM glucose, 1 mM pyruvate, and 2 mM L-Glutamine, and subjected to different treatments of Ang-II, SCFAs, and/or HO-2 inhibitor, as indicated above. At 18 h post-treatment, a mitochondrial stress test was performed in a Seahorse Bioscience XF96 analyzer with sequential additions of oligomycin A, FCCP, and antimycin/rotenone at 1, 1.5, and 2 μM each, respectively. The ATP-dependent oxygen consumption rate (OCR) was calculated by subtracting the OCR after the addition of oligomycin A from the baseline OCR and the basal extracellular acidification rate (ECAR) was measured prior to the addition of glucose.

### 2.12. Statistical Analysis

Data are expressed as mean ± SEM and were analyzed using GraphPad Prism 9.0 software. All data sets were analyzed for normality and equal variance. Kruskal–Wallis test and Dunn’s post hoc test were used for data sets where normal distribution could not be assumed. Two-tailed unpaired Student’s *t*-test and one-way ANOVA, followed by Tukey’s multiple comparison tests, were used for data sets with normal distribution. Two-way ANOVA followed by Tukey’s multiple comparison tests were used for grouped data sets. A *p*-value <0.05 was considered significant.

## 3. Results

### 3.1. SCFAs Reverse Ang-II-Induced Downregulation of HO-2

**Figure 1:**
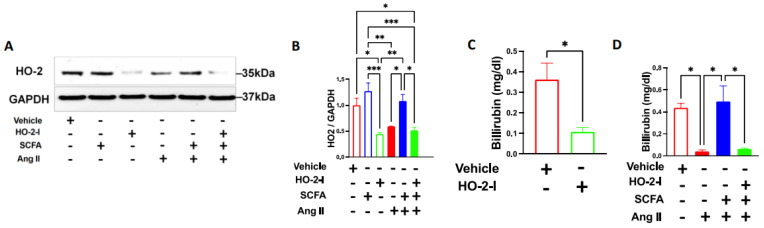
**SCFAs rescue HO-2 after Ang-II treatment.** Immunoblots (**A**), quantifications (**B**), and bilirubin assay (**C**,**D**) showing that the Ang-II-induced reduction in HO-2 protein expression and activity in brain microvascular endothelial cells was rescued by the SCFAs/HO-2 axis. * *p* < 0.05; ** *p* < 0.01; *** *p* < 0.001. N = 3–4. SCFAs: short-chain fatty acids; Ang II: angiotensin II; and HO-2 I: heme oxygenase 2 inhibitor.

To examine whether SCFAs regulate HO-2 during Ang-II treatments, we analyzed the HO-2 expression level and activity in HBMECs subjected to Ang-II in the presence or absence of SCFAs. Decreased levels of HO-2 expression were observed in HBMECs following Ang-II treatment compared to the vehicle (Figure 1A,B). Interestingly, in the presence of SCFAs, HBMECs exhibit fully restored HO-2 expression when compared to vehicle-treated cells (Figure 1A,B). Next, we tested the activity of HO-2 by measuring the bilirubin levels. Ang-II significantly decreased bilirubin levels which were recovered with SCFAs co-treatment (Figure 1C,D). These results suggest that SCFAs exert a regulatory effect on HO-2 expression and activity.

### 3.2. SCFAs Improve Ang-II-Induced Endothelial Dysfunction by Regulating HO-2

**Figure 2:**
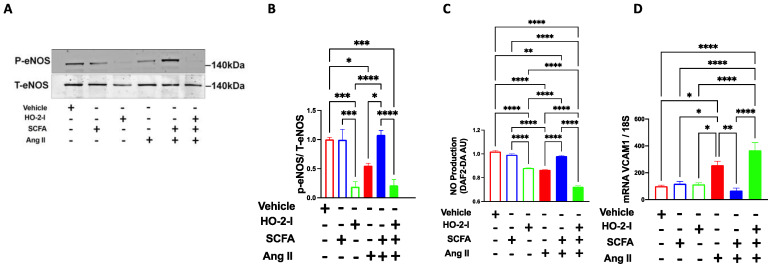
**SCFAs rescue Ang-II-induced eNOS reduction through HO-2.** Immunoblots (**A**) and quantification (**B**), NO production assay (**C**), and qPCR for VCAM1 (**D**) showing that the Ang-II-induced reductions in eNOS expression and NO production and increased VCAM1 expression in brain microvascular endothelial cells were reversed by the SCFAs/HO-2 axis. * *p* < 0.05; ** *p* < 0.01; *** *p* < 0.001; **** *p* < 0.0001. N = 3–7. SCFAs: short-chain fatty acids; Ang II: angiotensin II; HO-2 I: heme oxygenase 2 inhibitor; eNOS: nitric oxide synthase; NO: nitric oxide; and VCAM1: vascular cell adhesion molecule.

To assess the effect of SCFAs on the Ang-II-induced endothelial dysfunction in vitro, HBMECs were exposed to Ang-II in the presence or absence of SCFAs, and the endothelial function markers were evaluated. A significant reduction in phosphorylated eNOS levels (Figure 2A,B) and NO production (Figure 2C) and an increased level of VCAM1 (Figure 2D) were observed in Ang-II-treated cells compared to the vehicle. These effects were fully reversed in the presence of SCFAs (Figure 2). Interestingly, pharmacological inhibition of HO-2 using an HO-2 inhibitor (HO-2 I, SnPP 5 μM) annulled the beneficial effects of SCFAs in vitro (Figure 2). These data indicate that the SCFAs reverse the Ang-II-induced endothelial dysfunction via an HO-2-mediated pathway.

### 3.3. SCFAs Reduce Ang-II-Induced Endothelial Inflammation by Regulating HO-2

**Figure 3:**
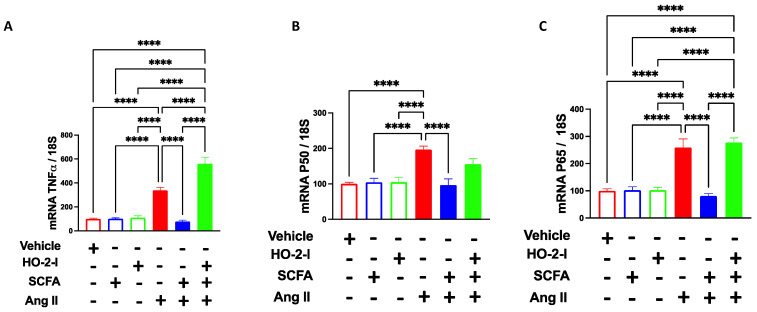
**SCFAs reduce Ang-II-induced inflammation by mediating HO-2.** qPCR for TNFα (**A**), p50 (**B**), and p65 (**C**) showing that the Ang-II-induced increases in these inflammatory markers in brain microvascular endothelial cells were reversed by the SCFAs/HO-2 axis. **** *p* < 0.0001. N = 7–8. SCFAs: short-chain fatty acids; Ang II: angiotensin II; HO-2 I: heme oxygenase 2 inhibitor; TNFα: tumor necrosis factor-alpha; and p50 and p65: NF-κB subunits.

Inflammation plays a detrimental role in regulating ECs and blood flow [25,26]. Along the same lines, the present study showed that HBMECs subjected to Ang-II displayed higher expression levels of TNFα, NFκB-p50, and NFκB-p65 (Figure 3) compared to vehicle-treated HBMECs. Similarly, co-treatment with SCFAs prevented the increase in these inflammatory markers, an effect that is abolished by the presence of the HO-2 inhibitor, indicating an intermediatory role of HO-2 in the SCFAs-mediated effect (Figure 3). 

### 3.4. The SCFAs/HO-2 Axis Regulates Calcium Homeostasis in Mitochondria from Cerebral ECs

**Figure 4:**
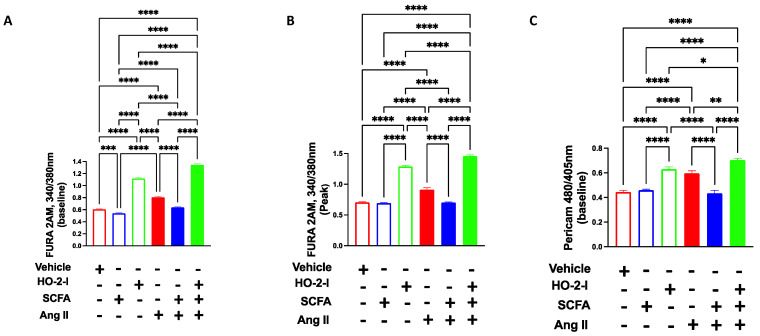
**SCFAs reduce Ang-II-induced Ca^2+^ increase by mediating HO-2.** Cytosolic Ca^2+^ at baseline (**A**), at peak (**B**), and mitochondrial Ca^2+^ (**C**) showing that Ang-II-induced increases in Ca^2+^, in brain microvascular endothelial cells were reversed by the SCFAs/HO-2 axis. * *p* < 0.05; ** *p* < 0.01; *** *p* < 0.001; **** *p* < 0.0001. N = 3–5. SCFAs: short-chain fatty acids; Ang II; angiotensin II; HO-2 I: heme oxygenase 2 inhibitor; and FURA 2AM and Pericam: Ca^2+^ trackers.

HO-2 activity is closely regulated by cellular calcium during neuronal activity [27]. To test whether this relationship exists between the SCFAs/HO-2 axis and mitochondrial calcium homeostasis, we evaluated the cytosolic and mitochondrial calcium levels following exposure to Ang-II and in the presence or absence of SCFAs. At one-day post-Ang-II treatment, HBMECs exhibited increased cytosolic and mitochondrial Ca^2+^ levels compared to vehicle-treated cells (Figure 4). Interestingly, SCFAs co-treatment normalized both cytosolic and mitochondrial Ca^2+^ levels, an effect that was abolished in the presence of HO-2 I (Figure 4).

### 3.5. SCFAs Normalized Mitochondrial Membrane Potential by Mediating HO-2 following Ang-II Treatment

**Figure 5:**
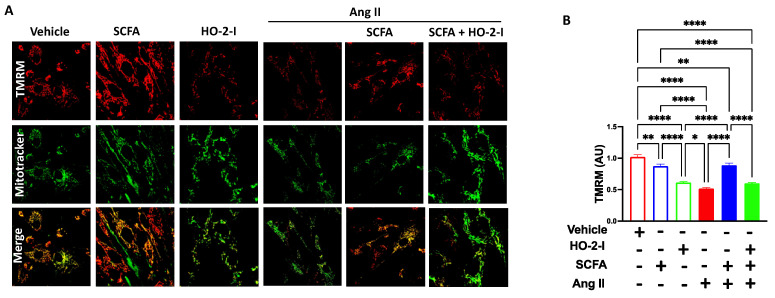
**SCFAs rescued Ang-II-induced membrane potential damage by mediating HO-2.** TMRM fluorescence integrated density (**A**) and representative images (**B**) showing that Ang-II-induced membrane potential hypopolarization in brain microvascular endothelial cells was reversed by the SCFAs/HO-2 axis. * *p* < 0.05; ** *p* < 0.01; **** *p* < 0.0001. N = 3–5. SCFAs: short-chain fatty acids; Ang II: angiotensin II; HO-2 I: heme oxygenase 2 inhibitor; and TMRM: Tetramethyl rhodamine, Methyl Ester, Perchlorate.

Mitochondria utilize the electrochemical potential across their inner membrane (ΔΨm) to stimulate mitochondrial Ca^2+^ entry and promote _mt_ROS production, in part, by OXPHOS activity stimulation. In the current study, we investigated whether the activation of the SCFAs/HO-2 axis influenced ΔΨm following Ang-II treatment. Our data, using the TMRM fluorescence, showed that ΔΨm was hyperpolarized following Ang-II treatment. While the treatment with only SCFAs was able to preserve membrane potential, the combination with HO-2 I abolished this effect (Figure 5).

### 3.6. The SCFAs/HO-2 Axis Regulates Mitochondrial ROS, H_2_O_2_ and Mitochondrial Function

**Figure 6:**
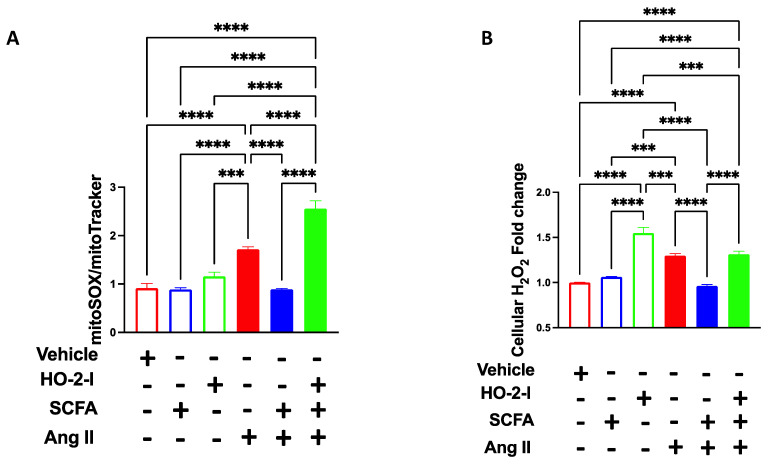
**SCFAs rescued Ang-II-induced mitochondrial ROS and H_2_O_2_ by mediating HO-2.** MitoSOX fluorescence integrated density (**A**) and H_2_O_2_ production (**B**) showing that Ang-II-induced increases in mitochondrial oxidative stress in brain microvascular endothelial cells were reversed by the SCFAs/HO-2 axis. *** *p* < 0.001; **** *p* < 0.0001. N = 3–5. SCFAs: short-chain fatty acids; Ang II: angiotensin II; HO-2 I: heme oxygenase 2 inhibitor; mitoSOX: dye for mitochondrial superoxide; and H_2_O_2_: hydrogen peroxide.

HO-2 has been shown to possess an antioxidant effect in the cerebrovascular endothelium [28]. To test whether SCFAs will prevent Ang-II-induced mitochondrial oxidative stress via the HO-2 pathway, we measured mitochondrial (mt)ROS production and its byproduct, cellular H_2_O_2_. Ang-II treatment increased the level of Mito-SOX fluorescence intensity, indicating higher mitochondrial ROS production; the co-treatment with SCFAs abolished Ang-II-induced mtROS, whereas the presence of HO-I annulled the positive effect of the SCFAs (Figure 6A). Since most of the ROS produced by mitochondria are rapidly converted to H_2_O_2_ by manganese superoxide dismutase (MnSOD), we measured the levels of H_2_O_2_ in cultured HBMECs using the Amplex Red assay. The results showed that the treatment with SCFAs reduced the H_2_O_2_ levels induced by the Ang-II treatment (Figure 6B). This effect was abolished in the presence of the HO-2 inhibitor.

### 3.7. SCFAs Rescued Ang-II-Induced Mitochondrial Respiration Damage by Mediating HO-2

**Figure 7:**
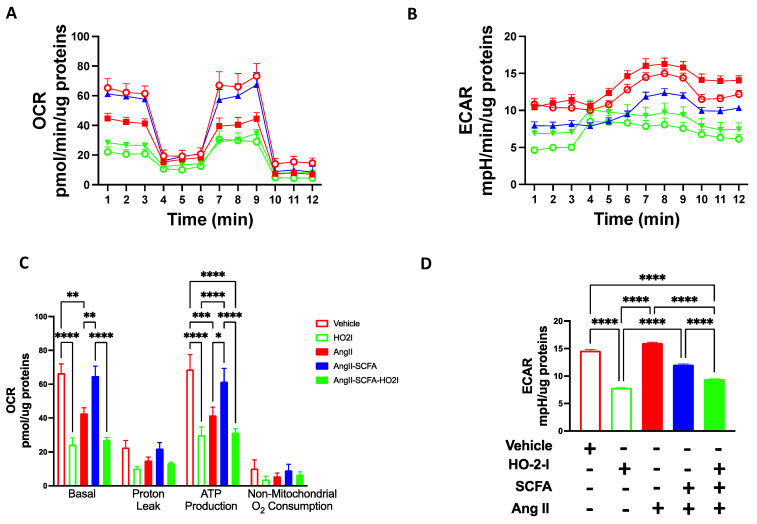
**SCFAs rescued Ang-II-induced mitochondrial respiration impairment.** OCR (**A**,**C**) and ECAR (**B**,**D**) show that Ang-II-induced changes in OCR and ECAR in brain microvascular endothelial cells were reversed by the SCFAs/HO-2 axis. * *p* < 0.05; ** *p* < 0.01; *** *p* < 0.001; **** *p* < 0.0001. N = 50,000 cells per well/four different experiments. SCFAs: short-chain fatty acids; Ang II: angiotensin II; HO-2 I: heme oxygenase 2 inhibitor; OCR: oxygen consumption rates; ECAR: extracellular acidification rate.

The same pattern was observed in oxygen consumption using a Seahorse stress test (Figure 7A). While a decreased oxygen consumption rate (OCR) was observed in the presence of Ang-II, HBMECs exhibited an increased extracellular acidification rate (ECAR) following Ang-II treatments (Figure 7B), indicating a metabolic switch to glycolysis. This phenotype was reversed in the presence of SCFAs. Treatment with the HO-2 inhibitor abolished the beneficial effect of SCFAs on OCR levels following Ang-II treatments (Figure 7). Interestingly, the ECAR levels completely declined in the presence of the HO-2 inhibitor indicating other cytosolic pathways where HO-2 potentially participate to maintain metabolic activity.

## 4. Discussion

Overall, this study showed that HO-2 expression and activity are altered during cellular stress. Additionally, the reduction in HO-2 expression and activity in cerebrovascular endothelial cells causes mitochondrial and endothelial dysfunction. SCFAs were able to restore the level of HO-2 and therefore rescued the mitochondrial and endothelial function.

The relationship between HO-1 and Ang-II-induced hypertension has been well documented in the literature [29]. Indeed, HO-1 levels significantly decreased in response to Ang-II, and HO-1 overexpression reversed the detrimental effect of Ang-II on vascular function and blood pressure [29,30]. However, little is known about the relationship between Ang-II and HO-2. HO-2 possesses cytoprotective effects due to its antioxidant, antiapoptotic, and anti-inflammatory effects [15]. Here, we showed evidence that Ang-II was able to reduce the level and activity of HO-2 in primary HBMECs. The current data demonstrate that HO-2 plays an important role in regulating HMBEC function during hypertensive conditions.

Hypertension is associated with reduced levels of SCFAs [31,32]. While several studies demonstrated the protective role of SCFAs during hypertension, via normalizing blood pressure and vascular reactivity [32,33,34], it remains difficult to dissect whether the effect of SCFAs on vascular endothelial cells was direct or a consequence of blood pressure reduction. Although there are studies that provide convincing evidence that SCFAs, through a direct or indirect mechanism, can activate HO-1 [35], there have been no studies to date that have evaluated the effect of SCFAs on HO-2. In the present study, we evaluated the direct effect of SCFAs on HBMECs treated with Ang-II in vitro. SCFAs supplementation was able to recover HO-2 expression and activity following Ang-II treatment. Since HO-2 is involved in many cytoprotective pathways [15], we speculate that SCFAs, by acting on HO-2, will positively impact cellular function under stress.

Typically, Ang-II induces endothelial dysfunction by reducing NO and increasing the proinflammatory markers and adhesion molecules [36]. Our data are in accordance with these observations since we showed a reduction in NO production and an increase in inflammatory markers and adhesion molecules in HBMECs following Ang-II exposure. Co-treatment with SCFAs reversed these effects. The beneficial effect of SCFAs on endothelial function such as the increase in NO production [36], anti-inflammatory effects [37,38], and the reduction in adhesion molecules [39] is very well established. However, the exact mechanism by which SCFAs exert this beneficial effect, especially in HBMECs is lacking. In the present study, we have evidence that SCFAs’ beneficial effects on HBMECs were achieved through HO-2. The outcome of the study and the data are a proof of concept that the SCFAs/HO-2 axis is a key determinant of endothelial function.

The relationship between HO-1 and mitochondrial function is well-documented [13,14]. However, little is known about the relationship between HO-2 and mitochondrial function, especially in cerebrovascular endothelial cells. A recent study showed that similar to HO-1, HO-2 can translocate to the mitochondria [16]. However, the role of HO-2 in the mitochondria remains largely unknown. The present data shows that HO-2 is a key component for mitochondrial function as it regulates mitochondrial Ca^2+^ homeostasis, membrane potential, mitochondrial ROS, H_2_O_2_, and oxygen consumption. Furthermore, SCFAs are known to regulate mitochondrial function in the gut [40], lymphoblastoid cells [41], hepatocytes [42], beta cells [5], and adipose tissue [43]. Nonetheless, the relationship between SCFAs and cerebrovascular endothelial cells is not known, and our data revealed a novel mechanism by which SCFAs regulate mitochondrial function in HBMECs. Our studies demonstrated that SCFAs, by increasing HO-2, improve mitochondrial function during stress, such as exposure to Ang-II.

Fecal SCFA levels were shown to play an important role in reducing the body weight of high-fat diet-induced obese mice (HFD) [6]. Additionally, treatment with exogenous acetate, propionate, or butyrate has been shown to prevent weight gain in HFD mice and overweight humans [44,45]. These findings provide insights into new targeting mechanisms of SCFAs, which may be important for preventing or treating obesity-induced cerebrovascular diseases.

Our study has shed light on a new pathway by which SCFAs could affect mitochondrial function in HBMECs during stress through the regulation of HO-2. SCFAs are known to be affected by hypertension [46] and neuropathological diseases such as Alzheimer’s disease [47]. Thus, examining this mechanism in vivo using a disease model known to produce gut dysbiosis-induced alteration in SCFAs levels, such as hypertension or obesity, will support a translational pipeline connecting SCFAs to cerebrovascular function.

**Clinical significance:** In recent years, there has been a growing body of evidence supporting the role of gut bacteria, which plays a pivotal part in the regulation of the onset and progression of cerebrovascular diseases. Benakis et al. demonstrated that gut dysbiosis occurs in several animal models of ischemic stroke. Specifically, their studies exhibit that gut microbiota can regulate neuroinflammatory responses and thereby influence brain recovery [48]. The data from this study highlights the delicate play between the brain and gut microbiome following acute brain injury. Additionally, Xiong et al. highlight the taxonomic and functional bacteria changes between patients with intraparenchymal hemorrhage compared to healthy individuals [49]. This data strongly supports the hypothesis that gut microbiota is a target of intracerebral hemorrhage-induced systemic alteration. Consequently, gut dysbiosis could have a substantial impact on the outcome of intracerebral hemorrhages and establishes the connection between gut microbiome health and cerebrovascular perfusion. Furthermore, the gut microbiota has been shown to contribute to cerebral small vessel disease [50], and the pathophysiology of cranial aneurysms by modulating inflammation [51]. It is important to note that gut microbiota (gut dysbiosis) has not only been linked to several cerebrovascular conditions such as ischemic stroke, intracerebral hemorrhage, intracranial aneurysm, and cerebral microvascular disease but also to diseases that impact the cerebrovascular physiology such as obesity and hypertension. Although the influence of gut microbiota on obesity and hypertension has been extensively studied, less is known about the effect of gut dysbiosis on obesity-induced cerebrovascular diseases. The gut microbiota communicates with the brain through its metabolites. Several studies have shown that bacterial metabolite profiles were altered in patients with various brain diseases [52]. SCFAs are gut microbiota-derived metabolites that regulate the gut–brain axis and are speculated to impact the cerebrovascular physiology following gut dysbiosis. It has been shown that SCFA are involved in neurodegenerative diseases including Alzheimer’s [53], Autism [54], and Parkinson’s [55]. Additionally, SCFAs show effectiveness in improving post-stroke recovery via an immunological mechanism [56]. The exact mechanism by which SCFAs affect cerebrovascular physiology is yet to be determined. In our in vitro study, we elucidate a potential mechanism by which SCFA could influence cerebrovascular physiology. We show that SCFA was able to restore the level of HO-2 and therefore rescue the cerebral mitochondrial and endothelial function. Extrapolating this data to an in vivo model of cerebrovascular disease is of great clinical significance since it could be a key step in developing novel therapeutic targets to treat central nervous diseases. Furthermore, our results provide a framework for molecular studies to better characterize the molecular mechanisms of SCFAs.

## Data Availability

Data are available upon request.
